# Longitudinal radiographic behavior of accessory navicular in pediatric patients

**DOI:** 10.1007/s11832-016-0777-x

**Published:** 2016-11-02

**Authors:** Derrick M. Knapik, Sahejmeet S. Guraya, Keegan T. Conry, Daniel R. Cooperman, Raymond W. Liu

**Affiliations:** 1 Department of Orthopaedic Surgery, University Hospitals Cleveland Medical Center, Cleveland, OH USA; 2 Rainbow Babies and Children’s Hospital, School of Medicine, Case Western Reserve University, 11100 Euclid Ave., Cleveland, 44106 OH USA; 3 Department of Orthopaedic and Rehabilitation, Yale University, New Haven, CT USA

**Keywords:** Accessory navicular, Ossicle, Pediatric, Foot, Fusion

## Abstract

**Background:**

An accessory navicular is generally asymptomatic and discovered incidentally on radiographs. The natural history of an accessory navicular in the pediatric population is largely undescribed.

**Methods:**

The medical charts of 261 pediatric subjects undergoing 2620 annual unilateral radiographs of the foot and ankle (age range 0.25–7 years at enrollment) were reviewed. Radiographs were examined to determine the incidence of accessory navicular, with focus on the age at appearance and, if present, the age at fusion. Skeletal maturity was graded based on ossification pattern of the calcaneal apophysis.

**Results:**

Accessory navicular was identified in 19 subjects (*n* = 12 males, *n* = 7 females, *p* = 0.43), appearing significantly earlier in the female subjects than in the male ones (*p* = 0.03). Fusion was documented in 42% (*n* = 8) of subjects, occurring at a mean (±standard deviation) age of 12.5 ± 1.0 years in females and 14.1 ± 2.7 years in males. Skeletal maturity grading demonstrated comparable stages of maturity at the time of fusion between male and female subjects (*p* = 0.5). Based on an analysis of 160 subjects with serial images extending at least one standard deviation past the mean age of appearance, the overall incidence was 12%.

**Conclusion:**

Our review of pediatric subjects showed that accessory navicular appeared earlier in females than in males. Fusion occurred in 42% of patients at comparable levels of skeletal maturity between the male and female subjects. No significant differences in overall incidence, skeletal maturity, fusion rate, or age of fusion were noted between the male and female subjects.

## Introduction

The accessory navicular, also known as “os tibiale externum”, “os navicularum,” or “prehallux”, represents a developmental variant within the foot appearing secondary to failed fusion from a secondary ossification center off the navicular [1–5]. Typically found on the posteromedial aspect of the foot, existing adjacent or completely separated from the navicular, the accessory navicular is one of the most commonly identified ossicles within the interior of the foot in pediatric and adult patients [1, 4, 6]. The majority of accessory naviculars are asymptomatic and discovered incidentally following unrelated foot or ankle trauma [1, 7–9]. However, the accessory navicular can be a source of foot pain following overuse or trauma [10, 11], necessitating conservative management, and in rare cases, operative intervention [7, 12, 13].

Skeletally immature pediatric patients possess the highest likelihood of foot pain secondary to accessory navicular [4, 14–16]. Pain generally presents in the adolescent athlete who complains of chronic medial-sided foot pain, limiting activity due to worsening symptoms with weight bearing [7, 17]. However, few studies have examined the incidence of the accessory navicular in an exclusively pediatric population; instead, most studies have combined results from pediatric and adult patients [3]. Furthermore, in the pediatric population, little is known regarding the longitudinal behavior of the accessory navicular over time in regards to fusion rate.

The purpose of this study was to analyze a longitudinal collection of healthy children with annual radiographs of the foot and ankle to better understand the natural history of the accessory navicular. Specifically, we sought to determine: (1) the incidence of accessory navicular in a healthy pediatric population; (2) the mean age of accessory navicular appearance, age of fusion and fusion rates; (3) the timing of fusion in relation to the degree of skeletal maturity based on the ossification pattern of the calcaneus.

## Materials and methods

Digitized anterior–posterior and lateral radiographs of the left foot of 261 children (*n* = 142 males, *n* = 119 females) from the Bolton-Brush Growth Study Center were screened. This database contains radiographs longitudinally collected from healthy Caucasian children growing up in Cleveland, Ohio from 1929 to 1942, allowing for the study of osseous growth during adolescence. All subjects included into the study were children identified by teachers and physicians as exemplifying the healthy, normally developing child. Children included within the study were longitudinally followed throughout growth. After age 5 years they underwent annual medical evaluations which included obtaining radiographs of the skull, chest, pelvis and left shoulder, wrist, hand, knee, tibia, and foot/ankle. In total, more than 250,000 radiographs and 22,000 physical examinations in over 4000 children were obtained during the study period [18]. The authors utilized all available radiographs from a sample of the collection that have been previously digitally scanned and optimized for evaluation of bony anatomy. Of note, the Bolton-Brush database is the same historical collection used to establish the Greulich and Pyle bone age atlas. Prior to data collection, approval was obtained by the authors’ Institutional Review Board. Original radiographs from the collection have been digitized and enhanced (Adobe Photoshop CC, 2015; Adobe Systems Inc., San Jose, CA) to allow for easy access and examination. Two authors inspected all available foot and ankle radiographs from 261 subjects, with a minimum of four annual radiographs captured between ages 3 months and 17 years. Due to the age of the radiographs (approx. 80 years), a small number of radiographs (*n* = 22 images) were excluded from subjects within the cohort secondary to the inability to fully visualize the relevant anatomy following digital optimization. Subjects spanning the full range of child development were studied to fully understand the longitudinal nature of accessory navicular development, although only subjects with follow-up images at least one standard deviation (SD) beyond the average age of appearance were used to calculate incidence.

In total, 2620 radiographs were separately evaluated for the presence of accessory navicular, noting the age of first appearance and the age of ossicle fusion, if applicable. Results were then compared, and radiographs without consensus on the presence or absence of a true accessory navicular were evaluated by the senior author to determine if a true ossicle was present. For all subjects found to have an accessory navicular, their study charts were examined to determine the presence of symptoms related to foot pain that may be attributed to the presence of a symptomatic accessory navicular. For each subject, the shortest distance from the ossicle to the navicular was measured at the time of presentation. In addition, the width and height of each ossicle at the time of presentation was measured and the area calculated using an ellipse as a model.

Skeletal maturity was graded by ossification pattern of the calcaneus on lateral radiographs and scored by two authors using the maturity grading system designed by Nicholson et al. [19]. The classification is divided into six distinct stages: Stage 0, no ossification; Stage 1, ossification of the calcaneus <50% of the metaphysis; Stage 2, apophyseal ossification >50% without fully covering the plantar surface; Stage 3, apophyseal extension over the plantar surface and within 2 mm of the calcaneal concavity; Stage 4, evidence of initial fusion between the apophysis and the metaphysis, Stage 5, complete fusion.

### Statistical analysis

Intraclass correlation coefficients (ICCs) were calculated between grades using the SPSS statistical package (IBM Corp., Armonk, NY). Following established recommendations, an ICC of <0.4 was determined to be poor, 0.4–0.75 to be fair to good, and >0.75 to be excellent [20, 21]. Differences in the incidence of accessory navicular and fusion rates between male and female subjects were compared by Chi-square test, while the age of fusion was analyzed using an unpaired Student’s *t* test.

## Results

Of the 261 subjects screened (*n* = 2620 radiographs), an accessory navicular was identified in 19 subjects (12 males, 7 females). No significant difference in accessory navicular incidence was present between males and females (*p* = 0.43). Accessory navicular appeared significantly earlier in females (mean ± SD: 10.1 ± 0.7 years) than males (12.2 ± 2.2 years) (*p* = 0.03) (Table 1). A total of 39% of subjects (*n* = 51 female, *n* = 50 male) did not have radiographs one standard deviation beyond the mean age of accessory navicular appearance for females (12 years) or males (14 years). Based on the remaining 160 subjects, the adjusted incidence of accessory navicular was 12%. Review of the study charts of patients with evidence of accessory navicular revealed that no subject had any complaints of pain within the mid-foot.

**Table 1:** Accessory navicular characteristics in pediatric cohort

Sex	Mean age of appearance (years)^a^	Adjusted incidence	Fusion rate	Mean age of fusion (years)^b^
Male	12.2 ± 2.2	13% (12/92)	33% (4/12)	14.1 ± 2.7
Female	10.1 ± 0.7	10% (7/68)	57% (4/7)	12.5 ± 1.0
Male + female	11.4 ± 2.0	12% (19/160)	42% (8/19)	13.3 ± 2.1

Values in table are presented as the mean ± standard deviation (SD) or as a percentage with the numbers in parenthesis, as appropriate

^a^Males vs. females: difference is significant at *p* = 0.03

^b^There were no significant differences in the age of fusion between males and females (*p* = 0.80)

**Fig. 1:**
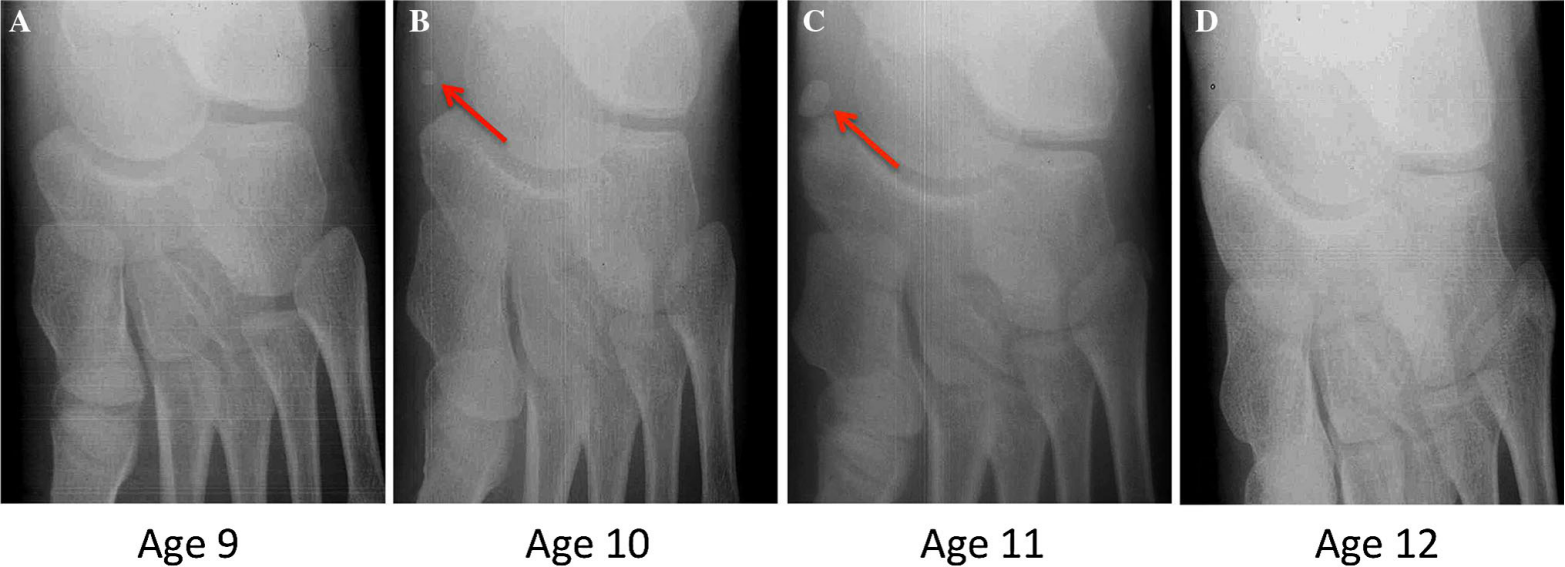
Serial radiographs of an asymptomatic male patient showing no radiologic evidence of accessory navicular (**a**), the initial appearance (**b**, *red arrow*), accessory ossicle growth with continued discontinuity (**c**, *red arrow*), and osseous fusion (**d**)

Fusion of the accessory navicular occurred in eight (42%) subjects with an equal distribution in males (*n* = 4) and females (*n* = 4) (*p* = 0.80) (Fig. 1a–d). The mean (±SD) overall age of fusion was 13.3 ± 2.1 years (females 12.5 ± 1.0 years; males 14.1 ± 2.7 years). No significant differences in the age of fusion was observed between males and females (*p* = 0.80), although this comparison was limited with just four patients in each group.

Accessory navicular fusion was not significantly associated with the distance from the ossicle to navicular (*p* = 0.97) or the mean area of the ossicle (*p* = 0.52) at the time of presentation (Table 2).

**Table 2:** Differences in ossicle gap and area at the time of presentation

Fusion/no fusion	Mean ossicle to navicular gap (mm)	*p* value	Mean ossicle area (mm^2^)	*p* value
Fusion	1.2 ± 1.5		88 ± 91	
No Fusion	1.2 ± 1.0	0.97	63 ± 65	0.52

Values in table are presented as the mean ± SD

Grading of skeletal maturity based on the pattern of calcaneal ossification produced an excellent interobserver ICC of 0.94 with comparable stages at the time of fusion between male (mean ± SD: 3.8 ± 1.3) and female (4.3 ± 0.5) subjects (*p* = 0.5). Of all eight subjects with evidence of ossicle fusion, 88% (*n* = 7/19) underwent fusion at or within 1 year of reaching Stage 4. Of the 11 subjects without fusion, 73% (*n* = 6/8 males,* n* = 2/3 females) were followed to Stage 5.

## Discussion

The accessory navicular is rarely symptomatic and generally represents a developmental aberration within the ankle of pediatric and adult patients. As a result, the true incidence in an asymptomatic population cannot be extrapolated in the absence of a broad screening radiologic evaluation. In this study, 7% (*n* = 19/261) of the overall cohort was found to have evidence of an accessory navicular, with a higher and more realistic rate of 12% when only those with a follow-up one standard deviation beyond the average age of appearance were considered.

Previous investigations have demonstrated no consistent differences in the incidence of accessory navicular in adult or pediatric patients based on sex. Within this cohort, comparable incidence rates were present in both males and females. Similar incidence studies by Coskun et al. identified accessory navicular at a similar rate in asymptomatic females (*n* = 65, 6.6%) and males (*n* = 51, 5.2%) [1], while Huang et al. found a slight difference in symptomatic females (*n* = 186/835, 22.2%) and males (*n* = 143/790, 18.1%) [3]. In contrast, Kruse et al. found that in asymptomatic adults, accessory navicular was significantly more common in females than males (*p* < 0.05), however, they did not report specific numbers [14]. While these previous studies were largely dependent on the rate of incidental discovery or presentation to clinicians for medial foot pain, the results from our study corroborate the findings of these other investigations in showing no large predilection for accessory navicular in the pediatric population based on sex. In addition, radiologic appearance of accessory navicular was found to occur significantly earlier in females than in their male counterparts. This observation is consistent with well-known developmental patterns, with females generally undergoing skeletal ossification an average of 2 years earlier than males [22].

Measurement of the amount of remaining skeletal growth during adolescence may be used to determine timing of accessory navicular fusion. Nicholson et al. [19] investigated the ossification of the calcaneal apophysis to quantify skeletal maturity in relation to the peak height velocity (PHV), better known as the adolescent “growth spurt” [23, 24]. Apophyseal ossification has been shown to follow a consistent and reproducible pattern, beginning at the calcaneal ossification center and gradually moving towards the dorsal and plantar surfaces in six distinct stages, which is conserved in both males and females [19]. Males and females have been found on average to undergo fusion around Stage 4, which is when partial fusion of the calcaneal apophysis has occurred. Based on the measured timing of the PHV versus calcaneal score, fusion occurs in females at a mean of 0.70 years after PHV and in males 0.67 years after PHV.

The timing of accessory navicular fusion may be a useful marker for clinicians when deciding upon treatment strategies for pediatric patients with symptomatic accessory navicular. While no standard treatment guidelines currently exist regarding indications for conservative versus operative management for symptomatic accessory navicular [25], most authors advocate for an initial course of conservative management [6, 26]. Given the timing of accessory navicular fusion shown in this study, one might more strongly favor conservative management if a child has not reached Stage 5 calcaneal ossification, signified by complete fusion of the apophysis.

This study was not without limitations. A sampling bias was present as not all subjects had radiographic films throughout their entire adolescence. We helped correct for this by calculating incidence rates for the entire cohort, as well as for the patients with follow-up beyond one standard deviation from the average age of appearance. The authors anticipate that some patients might have underwent fusion at a later time not captured within the radiologic databank due to the absence of films or poor quality. However, <1% of the radiographs (22 of 2642 images) were excluded from subjects within the cohort because of the inability to fully visualize the relevant anatomy due to image quality. In addition, unless specifically addressed in the medical charts, we were unable to determine if patients with symptomatic foot pain underwent any operative intervention in the years during and following data collection. Furthermore, our cohort consisted of subjects limited racially and geographically (middle and upper class Caucasian residents of Cleveland, Ohio from 1929 to 1942). As such, the results from this study are likely not representative of a more diverse general population. Lastly, this study does not provide information on the bilaterality of accessory navicular in pediatric subjects due to the method in which radiographs where collected, namely, only of the left foot and ankle only.

## Conclusion

In conclusion, this study demonstrates that in a largely asymptomatic pediatric patient population, accessory navicular was identified radiographically in 12% of patients with a follow-up of at least one standard deviation beyond the mean age of appearance. Accessory navicular was found to appear at a significantly earlier age in females than in males, while fusion occurred in 42% of subjects at an average of 2 years following appearance in both males and females. Fusion was also found to occur following PHV based on grading of calcaneal ossification growth patterns, with 88% of subjects undergoing fusion at or within 1 year of reaching Stage 4. Neither the distance from the ossicle to the navicular or the area of the navicular is predictive of eventual fusion. These data may be used to assist surgeons in treating pediatric patients with accessory navicular and to estimate whether a symptomatic child still has potential for fusion.
